# Adolescent Bariatric Surgery — Thoughts and Perspectives from the UK

**DOI:** 10.3390/ijerph110100573

**Published:** 2013-12-31

**Authors:** Marta Penna, Sheraz Markar, James Hewes, Alberic Fiennes, Niall Jones, Majid Hashemi

**Affiliations:** 1 UCLH Department of Weight Loss, Metabolic and Endocrine Surgery, University College London Hospital, 235 Euston Road, London NW1 2BU, UK; E-Mails: m.penna@doctors.org.uk (M.P.); sheraz_markar@hotmail.com (S.M.); 2 North Bristol NHS Trust, Beckspool Road, Frenchay, Bristol BS16 1JE, UK; E-Mail: jimhewes@hotmail.com; 3 St Anthony’s Hospital, 801 London Road, North Cheam, Surrey SM3 9DW, UK; E-Mail: alberic@agtwf.co.uk; 4 Barts and the London Children’s hospital, The Royal London Hospital, Whitechapel, London E1 1BB, UK; E-Mail: niallmartinjones@yahoo.co.uk

**Keywords:** adolescent obesity, bariatric surgery, opinions, healthcare professionals

## Abstract

Opinions of healthcare professionals in the United Kingdom regarding bariatric surgery in adolescents are largely unknown. This study aims to explore the perspectives of medical professionals regarding adolescent bariatric surgery. Members of the British Obesity and Metabolic Surgery Society and groups of primary care practitioners based in London were contacted by electronic mail and invited to complete an anonymous online survey consisting of 21 questions. Ninety-four out of 324 questionnaires were completed. 66% of professionals felt that adolescents with a body mass index (BMI) >40 or BMI >35 with significant co-morbidities can be offered surgery. Amongst pre-requisites, parental psychological counseling was chosen most frequently. 58% stated 12 months as an appropriate period for weight management programs, with 24% regarding 6 months as sufficient. Most participants believed bariatric surgery should only be offered ≥16 years of age. However, 17% of bariatric surgeons marked no minimum age limit. Over 80% of the healthcare professionals surveyed consider bariatric surgery in adolescents to be acceptable practice. Most healthcare professionals surveyed feel that adolescent bariatric surgery is an acceptable therapeutic option for adolescent obesity. These views can guide towards a consensus opinion and further development of selection criteria and care pathways.

## 1. Introduction

Adolescent obesity presents a potentially increasing source of clinical and economic strain to healthcare providers. The Health Survey estimates for England 2010 suggests a staggering 1.2 million schoolchildren aged between 2 and 15 years are overweight (≥*85th centile as per the UK 1990 growth reference chart*) and a further 1.3 million are obese (≥*95th centile*) [1]. The dramatic increase in the prevalence of childhood obesity since the 1970s [2,3] has been accompanied by a substantially increased risk of developing serious co-morbidities at an early age. Such co-morbidities include type 2 diabetes, hyperlipidemia, hypertension, sleep apnoea and cholelithiasis [4,5,6], as well as significant psychosocial consequences [7] and the increased likelihood of becoming obese adults [8]. Thus the clinical, psychological and economic consequences of adolescent obesity represent a significant challenge to healthcare systems across the world.

Several initiatives have been proposed and established to combat obesity through lifestyle changes. These include teaching children and parents healthy eating, encouraging more physical exercise and establishing weight management programs. In December 2006, the National Institute for Health and Clinical Excellence (NICE) provided the first national guidance on the prevention, identification, assessment and management of overweight and obesity in adults and children in England and Wales [9]. The guidelines recommend offering patients multi-modality interventions encouraging more physical activity and improved eating behaviour provided by a multidisciplinary team that includes general practitioners (GPs), pediatricians, bariatric dieticians and nurses, physiotherapists and psychologists. Drug therapy is advised only if there are severe physical or psychosocial co-morbidities, and similarly surgery is only recommended in exceptional circumstances.

Lifestyle interventions in overweight children have been shown to improve weight and cardio-metabolic outcomes, however, only modest changes are achieved when compared to those seen following surgery. Furthermore, the majority of studies collect data for up to 1 year from baseline and so ongoing research is required to determine the longer-term effectiveness of non-operative methods [10,11]. For these reasons bariatric surgery in adolescents represents an attractive option and is increasingly sought after by patients [12], seeking an effective and sustained remedy. To date, only a handful of studies from North America have explored healthcare professionals’ perspectives on adolescent bariatric surgery. These studies seem to suggest that despite the growing problem of childhood obesity and the potential difficulties encountered with sustaining weight loss using non-operative methods, many physicians are still reluctant to refer adolescents for a bariatric surgery assessment [13,14,15,16]. This is the first study completed in the United Kingdom (UK) to discover the current thoughts and beliefs of various healthcare professionals regarding adolescent bariatric surgery. The survey will provide valuable insights into bariatric practices throughout the UK and Ireland from the leading experts in this discipline. It will also form an important basis for the development of a longer-term consensus and policy in this rapidly evolving and challenging field of practice.

## 2. Methods

An online survey was constructed and distributed by electronic mail to members of the British Obesity and Metabolic Surgery Society (BOMSS) and a selection of GP practices across London, with a total of 324 healthcare professionals surveyed. Two subsequent mailings were sent to non-respondents at 3-week intervals. Paper questionnaires were also circulated and collected from London GP practices randomly selected by an online search of practices throughout the whole of London. The survey consists of 21 questions exploring healthcare professionals’ opinions about the management of obese adolescents and is based on key aspects surveyed in previous published studies and on the current NICE guidelines on the assessment and management of overweight and obese children (Appendix 1). Core themes explored:

(1) Prerequisites to adolescent bariatric surgery; which should be compulsory and which should be advisory.
(2) Minimum age and minimum body mass index (BMI) at which bariatric surgery should be considered.
(3) Any potential long-term benefits from bariatric surgery.
(4) Who should be involved in managing adolescent bariatric patients and length of post-operative follow up.

Results were tabulated and expressed as a percentage of the respondents.

## 3. Results and Discussion

A total of 94 out of 324 (29%) healthcare professionals completed the questionnaire. Of the respondents, 32% are general practitioners, 26% are bariatric surgeons, 18% bariatric dieticians, 9.5% pediatricians, 8.5% specialist bariatric nurses and the remaining 6% are endocrine, chemical pathologist or psychiatric physicians working in the United Kingdom (Table 1). The greatest response rate was achieved by the general practitioners, but this may reflect the fact that paper questionnaires were distributed to this group compared to just an online survey emailed to the rest of the healthcare professionals.

The vast majority of the respondents, around 89%, believe that the prevalence of adolescent obesity in the UK is increasing whilst 8.5% say it has reached a plateau and 2% are unsure. Only four out of the 45 physicians (9%) have ever referred an adolescent to be considered for bariatric surgery. 71% of bariatric surgeons that completed the survey have performed more than 40 bariatric procedures in adults; with gastric banding being the most commonly performed procedure, followed by Roux-en-Y gastric bypass and then sleeve gastrectomy. However, only five out of the 24 surgeons (21%) have performed bariatric surgery on adolescents in the previous 12 months (gastric banding or sleeve gastrectomy) and each surgeon performed no more than 10 cases.

**Table 1:** Demographics of respondents.

Specialty	Number (%)
**Surgeons**:	
Bariatric consultants	19 (20)
Bariatric clinical fellows	3 (3)
General surgery registrar	2 (2)
**Physicians**:	
General Practitioners	30 (32)
Pediatric consultants	9 (10)
Psychiatric consultant	3 (3)
Anaesthetic consultant	1 (1)
Endocrine consultant	1 (1)
Chemical pathologist	1 (1)
**Bariatric Specialist Nurses**	8 (9)
**Bariatric Specialist Dieticians**	17 (18)
**Total**	94 (100)

### 3.1. Compulsory Prerequisites to Adolescent Bariatric Surgery and Consent

**Figure 1:**
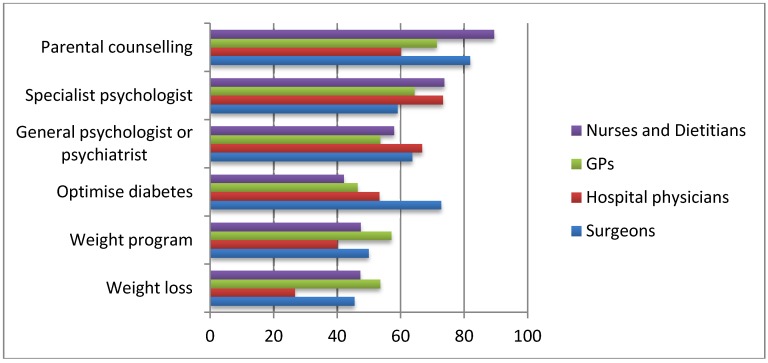
Compulsory prerequisites to bariatric surgery in adolescents during the assessment period.

Interestingly, the most frequently selected prerequisite to adolescent bariatric surgery was parental counselling and parental involvement in their child’s weight management; marked as a compulsory requirement by 91% nurses/dieticians, 82% of surgeons, 71% of general practitioners and 60% of hospital physicians (Figure 1). Assessment of the adolescent by a specialist psychologist or any general psychologist or psychiatrist was the second most popular pre-requisite selected.

Approximately 50% of respondents believe that adolescent should attend a monitored weight management program pre-operatively, with the majority, 58.4%, selecting up to 12 months as an adequate time period. It was also noted that almost 40% of surgeons felt that 6 months was a sufficient period for a weight management program, as opposed to only 17% of the physicians and nurses.

Less than 50% stated that pre-operative weight loss is essential. Optimization of diabetes mellitus separates the respondent groups with 73% of surgeons marking diabetic optimization as a compulsory prerequisite as opposed to only 44% of nurses/dieticians and 49% of physicians. With regards to consenting, the vast majority of the healthcare professionals feel that both parents and child should be actively involved in the process of consent (87.5% surgeons, 67% physicians, 57.1% nurses/dieticians). Just over 20% of the respondents believe that a child older than 16 years can give consent alone.

### 3.2. Minimum Age and BMI for Adolescent Bariatric Surgery

**Figure 2:**
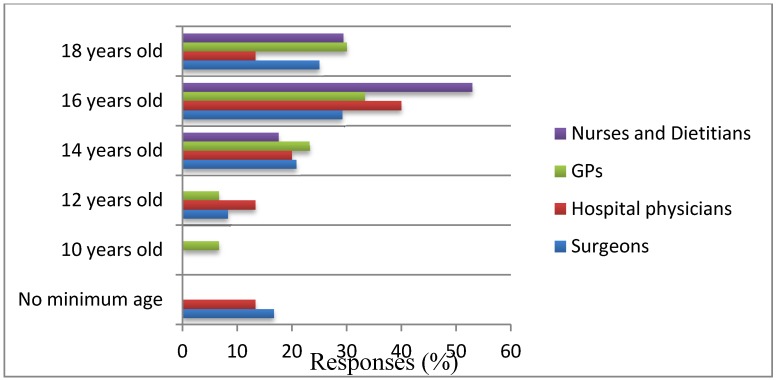
Minimum age for bariatric surgery.

Sixteen years of age was considered to be an acceptable minimum age for bariatric surgery by the majority of the respondents (Figure 2). Of note, 16.7% (four out of 24) surgeons believe that there should be no minimum age for bariatric surgery.

**Figure 3:**
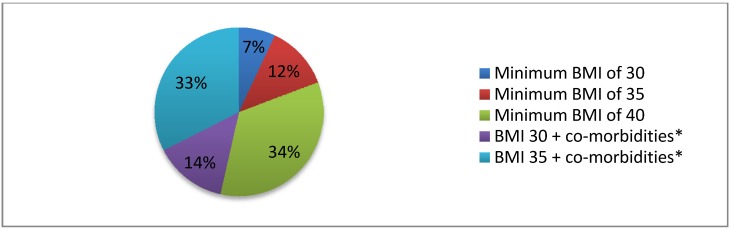
Minimum body mass index (BMI) for adolescent bariatric surgery; overall responses.

Note: * Co-morbidities include significant diseases that can be improved by losing weight such as type II diabetes mellitus and hypertension.

With regards to the minimum BMI above which bariatric surgery should be offered to adolescents the two options most commonly selected were a BMI of 40 and above, and/or a minimum BMI of 35 with other significant metabolic disease that can be improved by losing weight, such as type II diabetes mellitus and hypertension (Figure 3).

### 3.3. Potential Long-Term Benefits and Length of Follow up

A question posed was whether bariatric surgery would lead to long-term benefits, specifically in the following domains:

1. Reduced metabolic illness (such as diabetes mellitus and hypertension);
2. Reduced psychological distress;
3. Improve educational performance or,
4. no long-term benefit at all.

The majority of respondents (83% surgeons, 76% physicians, 76% nurses/dieticians) believe that bariatric surgery is likely to benefit the young patient by reducing the risk of either developing or ongoing metabolic illness long-term. More than 70% also believe that psychological wellbeing will improve post-surgery and some may achieve better educational performance too. Only 16% of all respondents do not believe in any long-term benefits from adolescent bariatric surgery. In terms of follow up, 74% of surgeons would review their patients lifelong, whilst 17% would see them for up to 5 years and 9% for only 1 year.

### 3.4. Team Members Involved in Managing Adolescent Bariatric Surgery Patients

A multidisciplinary team approach is felt to be most appropriate in managing obese adolescents undergoing bariatric surgery. Currently, some adolescent bariatric surgery is performed by adult bariatric surgeons, whilst some is performed by pediatric surgeons trained in bariatrics. The survey respondents were asked who should be performing such surgery on adolescents. Over 55% of each healthcare professional category surveyed believes that adult bariatric surgeons should receive training in treating adolescents. Views are more mixed when asked whether pediatric surgeons should gain experience in bariatric surgery, with only 12.5% of surgeons compared to 49% of physicians stating that pediatric surgeons should be trained in bariatrics.

Overall, the vast majority of the healthcare professionals (87.5% of surgeons, 77.3% of physicians, 85.7% of nurses/dieticians) that completed the survey consider bariatric surgery to be an acceptable treatment strategy in the treatment of adolescent obesity.

## 4. Discussion

The 21st century has seen an enormous rise in the prevalence of obesity, reaching epidemic proportions in both adults and children. Healthcare professionals from numerous different specialties have a critical role to play both preventing and treating obesity as well as managing the associated co-morbidities. This study is the first survey to be carried out in the UK that explores healthcare professionals’ opinions regarding adolescent bariatric surgery. The results from this survey show that the majority of the respondents believe that bariatric surgery should be offered to adolescents with either a minimum BMI of 40 or a BMI of 35 plus other associated co-morbidities which could be improved by losing weight; this reflects the current NICE guidelines. In contrast to a study published by Woolford *et al*. from the USA in which most participants thought patients should be at least 18 years old before being considered for surgery [13], healthcare professionals in our study believe 16 years to be an acceptable minimum age. In fact, up to 17% of bariatric surgeons selected no minimum age and, taken collectively, 35.6% of all respondents marked 14 years and younger as acceptable. Woolford’s study surveyed primary care pediatricians and family physicians, 46% of whom felt that the minimal age at which physicians would make a referral for bariatric surgery is 18 years old. In our study, a mixture of surgeons, GPs and hospital physicians were included and therefore likely to contribute to the difference in results. Hence, if we only look at the responses from GPs and physicians in our study group 34% perceive 16 years of age to be an acceptable minimum age for bariatric surgery, 24% selected 14 years and another 24% chose 18 years of age. Interestingly 25% of surgeons also selected 18 years as a minimum age but 17% believe that no minimum should be applied. Although our population group is very small and so firm conclusions cannot be drawn, the findings do highlight a divide in opinion amongst medical and surgical specialities with regards to age and whether physicians are more willing to refer younger patients for a bariatric surgical assessment, as bariatric surgery has become a more established specialty with promising results. A possible reason for such varied thoughts on age may be that skeletal maturity or the presence of co-morbidities and impact on the child’s life are more important determining factors than the patient’s actual age. These differences in minimum age for bariatric surgery between our study and the study by Woolford *et al.*, may also reflect social, cultural and legal differences regarding surgery, consent and perhaps the perception of decisional capacity that exist between the United States and United Kingdom.

A very important aspect of care highlighted by the results of this study is the involvement of parents in the management of adolescent obesity. Parental counselling was the most frequently selected compulsory prerequisite to surgery and parental involvement in the consenting process was deemed essential. Amongst our respondents, nurses, dieticians and surgeons were three groups particularly supportive of formal parental counselling. A strong parental bond and commitment is likely to have numerous benefits and promote a more successful outcome following surgery. As mentioned by Iqbal *et al*. [14], compliance is a crucial component in maintaining weight loss and requires education on general health and nutrition, determination and encouragement. Hence, parental input is extremely valuable, in providing emotional and psychological support to adolescent patients being evaluated for bariatric surgery. Further, a recent prospective study by Woodard *et al*., explored the “halo effect” for bariatric surgery in which it was noted that family members of patients that underwent bariatric surgery also appeared to lose weight and adopt a healthier lifestyle [15]. Given that children with obese parents are more likely to also be obese, and childhood obesity is strongly associated with the development of adult obesity, involvement of the whole family can potentially benefit all, and reduce the effects of this perpetuating cycle.

Interestingly, despite the fact that the majority of the respondents find bariatric surgery in adolescents an acceptable method of treatment only very few of them had ever referred an adolescent for a surgical assessment. Possible reasons may include the lack of available bariatric services locally or knowledge of such services, not encountering obese adolescent that may be possible candidates for surgery, patients’ refusal to be referred, or the physicians’ unwillingness to actually refer an adolescent. There is also the high bar set for referral for surgery by some of the available guidelines. The Scottish Intercollegiate Guidelines Network (SIGN) state that in most obese children (>98th centile) weight maintenance is an acceptable goal and that surgery can be considered for post pubertal adolescents with very severe to extreme obesity (BMI > 3.5 SD above the mean on 1990 UK charts) and severe co-morbidities [16].

Story *et al*., found that the commonly perceived barriers to obesity treatment included lack of patient motivation and family involvement and deficient support services [17]. Also, Iqbal *et al*., reported that reluctance to refer was most commonly due to the unknown long-term metabolic effects of weight loss surgery in such a young population [14]. However, there is growing evidence that early surgical intervention can lead to the resolution of obesity-associated co-morbidities such as type II diabetes and hypertension, as well as improving the psychosocial and educational consequences of morbid obesity [12,18,19,20]. Therefore it is possible that a low incidence of long-term metabolic side effects from bariatric surgery is outweighed by the disappearance of the weight-related co-morbidities [21,22,23]. The majority of respondents to our survey did feel that patients would obtain medical, psychological and educational benefits from bariatric surgery as adolescents, given the current status of medical knowledge.

Potential limitations of this study include the poor response rate of 29% and also that of restricting the general practice surgeries only to those in London, which therefore may not be representative of all family physicians in the UK. It may be that only those with a strong opinion answered the questionnaire whilst others with a more negative attitude or less interest were less likely to answer the questionnaire. Also, we enquired about healthcare professionals perceptions; actual referral patterns may differ significantly. Access and knowledge of bariatric services available and ease of referral are important influencing factors that were unable to be explored in this study.

## 5. Conclusions

Healthcare professionals in the UK are aware of the high prevalence of adolescent obesity and the co-morbidities associated with it. Potential benefits from bariatric surgery in adolescents are acknowledged including reduced metabolic illness, possible improved psychological and educational performance [24,25], however a number of specific criteria need to be met prior to consideration of surgical intervention. Parental involvement and counselling has been highlighted as a key component to the management of childhood obesity. Overall, the majority of the respondents considered bariatric surgery in adolescent an acceptable treatment modality. However, more robust data on the long-term complications of bariatric surgery in such a young population is still pending. The availability and ease of referral to specialized, multidisciplinary bariatric centres that serve adolescents in the UK is a subject that requires further investigation.

## Supplementary Files

Supplementary File 1 Supplementary (PDF, 65 KB)
