# Supplementary material for: Adolescent Bariatric Surgery — Thoughts and Perspectives from the UK
Source: Int J Environ Res Public Health. 2013 Dec 31;11(1):573–82. doi: 10.3390/ijerph110100573 (PMC3924461; doi:10.3390/ijerph110100573)
Supplement: Supplementary File 1 — Supplementary (PDF, 65 KB) [file ijerph-11-00573-s001.pdf]

Adolescent bariatric surgery: What do you think?

ADOLESCENT BARIATRIC SURGERY: WHAT DO YOU THINK?

University College London Hospital

Introduction

Dear Colleagues,

We would like to invite you to complete the enclosed questionnaire on the delivery of bariatric surgery for adolescents in the UK and Ireland. Almost two million school children in the United Kingdom are overweight, of whom about 700,000 are obese, according to the International Obesity Taskforce. Paediatricians and family practitioners are faced with making important treatment decisions for obesity in this age group. Current studies in North America show that despite the poor outcomes with non-operative methods of weight loss and the high satisfaction with the outcomes of bariatric procedures, physicians are still reluctant to refer children and adolescents for bariatric surgery.

Little is known about the opinions and referral behaviours of UK and Irish healthcare professionals regarding bariatric surgery in adolescents. The objective of our study is to explore the attitudes and perspectives of paediatricians, paediatric and bariatric surgeons, and other health professionals on adolescent bariatric surgery. The survey will provide valuable insights into bariatric practices throughout the UK and Ireland from the leading experts in this discipline. It will also form an important basis for the development of a longer-term consensus and policy in this rapidly evolving and challenging field of practice.

We greatly thank you for your time and thoughts and look forward to sharing the findings of the survey with you soon. All survey responses are anonymous.

1. Please fill in your details:

Profession:

Specialty:

Grade:

2. In your opinion, in the UK, is adolescent obesity:

- ◯ Increasing
- ◯ Decreasing
- ◯ Reached a plateau
- ◯ Unsure

Adolescent bariatric surgery: What do you think?

3. Have you ever referred an adolescent for bariatric surgery in the past?

- ◯ Yes
- ◯ No

4. If you practice bariatric surgery, how long for?

- ◯ Do not practice bariatric surgery
- ◯ Less than a year
- ◯ 1-2 years
- ◯ 3-5 years
- ◯ 6-9 years
- ◯ More than 10 years

5. Have you undertaken any training in bariatric surgery?

- ◯ Yes - if so please give details below
- ◯ No
- ◯ No as I am not a surgeon - Go to question 10

Details of bariatric surgery training:

6. How many ADULT bariatric procedures have you performed in the last 12 months?

- ◯ None - please go to question 8
- ◯ 1 to 10
- ◯ 11 to 20
- ◯ 21 to 30
- ◯ 31 to 40
- ◯ More than 40

7. Approximately what percentage of each procedure do you perform in ADULT patients?

	None performed	1 to 25%	26 to 50%	51 to 75%	76 to 100%
Roux en Y gastric bypass	◯	◯	◯	◯	◯
Sleeve gastrectomy	◯	◯	◯	◯	◯
Gastric banding	◯	◯	◯	◯	◯
Other - please specify	◯	◯	◯	◯	◯

Other (please specify)

8. How many ADOLESCENT bariatric procedures have you performed in the last 12 months?

- ◯ None - please go to question 9
- ◯ 1 to 10
- ◯ 11 to 20
- ◯ 21 to 30
- ◯ 31 to 40
- ◯ More than 40

9. Approximately what percentage of each procedure do you perform in ADOLESCENT patients?

	None performed	1 to 25%	26 to 50%	51 to 75%	76 to 100%
Roux en Y gastric bypass	◯	◯	◯	◯	◯
Sleeve gastrectomy	◯	◯	◯	◯	◯
Gastric banding	◯	◯	◯	◯	◯
Other - please specify	◯	◯	◯	◯	◯

Other (please specify)

Adolescent bariatric surgery: What do you think?

10. Which of these, if any, do you think should be compulsory prerequisites to bariatric surgery in adolescents during assessment? (Tick as many as you wish)

- [ ] Admission for a designated time to complete a monitored weight management program
- [ ] Psychological evaluation by any psychologist or psychiatrist with a special interest in eating disorders
- [ ] Psychological evaluation only by a specialized adolescent psychologist
- [ ] Optimisation of diabetic control accomplished prior to surgery
- [ ] Patient demonstrates successful weight loss
- [ ] Parental psychological counselling

11. Above which BMI do you think adolescent bariatric surgery should be offered?

- [ ] Minimum BMI of 30
- [ ] Minimum BMI of 35
- [ ] Minimum BMI of 40
- [ ] Patients with a minimum BMI of 30 and with other significant diseases that can be improved by losing weight (Type 2 diabetes mellitus, hypertension)
- [ ] Patients with a minimum BMI of 35 and with other significant diseases that can be improved by losing weight

12. Obese adolescents may attend monitored weight management programs. How long do you think patients should comply with such programs before surgery is offered?

- ○ Up to 6 months
- ○ 12 months
- ○ 2 years
- ○ Between 2 to 5 years
- ○ More than 5 years

Adolescent bariatric surgery: What do you think?

13. What do you think the minimum age for bariatric surgery should be?

- ◯ No minimum age
- ◯ 10 years old
- ◯ 12 years old
- ◯ 14 years old
- ◯ 16 years old
- ◯ 18 years old

14. In your opinion, who should give written consent for adolescent bariatric surgery?

- ◯ Only parents
- ◯ Both parents and child
- ◯ Children above 12 years of age with Gillick competence
- ◯ Children above 14 years of age with Gillick competence
- ◯ Children above 16 years of age with Gillick competence

15. How long do you routinely follow up bariatric patients?

- ◯ Not applicable
- ◯ Up to 6 months
- ◯ 1 year
- ◯ 5 years
- ◯ 10 years
- ◯ Lifelong

16. In your opinion, performing bariatric surgery in patients younger than 18 years will: (Tick as many as you wish)

- ☐ Reduce metabolic illness longterm
- ☐ Reduce psychological distress
- ☐ Improve educational performance
- ☐ None of the above

Adolescent bariatric surgery: What do you think?

17. Should adult bariatric surgeons be trained to treat adolescents?

- ◯ Yes
- ◯ No
- ◯ Not sure

18. Should paediatric surgeons be trained to do bariatric surgery?

- ◯ Yes
- ◯ No
- ◯ Not sure

19. In general do you consider bariatric surgery in adolescents:

- ◯ Acceptable
- ◯ Not acceptable

20. If you agree that there is a role for bariatric surgery in adolescents, who do you think should perform the surgery?

- ◯ Paediatric bariatric surgeons
- ◯ Adult bariatric surgeons
- ◯ A combination of both of the above within a multidisciplinary team
- ◯ Do not agree with bariatric surgery

21. Any further comments or thoughts

You have successfully completed the adolscent bariatric survey. Thank you very much!
